# Introducing the *Journal of the Medical Library Association*'s manuscript resubmission deadlines: creating accountability structures for our authors

**DOI:** 10.5195/jmla.2024.1902

**Published:** 2024-04-01

**Authors:** Alexander J. Carroll, Jill T. Boruff, Michelle Kraft

**Affiliations:** 1 alexander.j.carroll@vanderbilt.edu, Associate Director, Stevenson Science and Engineering Library, Vanderbilt University, Nashville, TN, United States; 2 jill.boruff@mcgill.ca, Co-Lead Editor, *Journal of the Medical Library Association*, Associate Librarian, Schulich Library of Physical Sciences, Life Sciences, and Engineering, McGill University, Montreal, QC, Canada; 3 kraftm@ccf.org, Co-Lead Editor, *Journal of the Medical Library Association*, Medical Library Director, Cleveland Clinic Foundation, Cleveland, OH, United States

## Abstract

The *Journal of the Medical Library Association* (*JMLA*) has made the decision to change our “revise-at-will” policy to instead adopt firmer deadlines for manuscript resubmissions. Beginning with this issue, manuscripts returned to authors with a “revise and resubmit” decision must be resubmitted within two months of the editorial decision. Likewise, manuscripts returned to authors with a “revisions required” decision must be resubmitted within one month of the editorial decision. This editorial discusses *JMLA*'s experience using a “revise-at-will” policy and outlines some anticipated benefits of the new resubmission deadlines.

One of the most frequent questions we receive from prospective *Journal of the Medical Library Association* (*JMLA*) authors is “how long will it take my manuscript to be published?” While we can provide rough estimates, our usual response is the deeply unsatisfying “it depends.”

Wendi Kaspar's 2016 editorial in College & Research Libraries presents a detailed account of the stages of each stage of manuscript review and preparation that is broadly representative of *JMLA*'s workflows [1]. While Kaspar provides average estimated days for each stage of the workflow that are roughly similar to *JMLA*'s, these approximations offered do not adequately convey the sizable standard deviations within these means. In our experience with manuscripts at *JMLA*, the time elapsed from submission to publication can vary from “several weeks” to “multiple years.” In other words, our time-to-publication data are not normalized, so reporting an average will not be a helpful guide for what prospective authors can expect.

As with other journals, the causes of delays in manuscript publication timelines at *JMLA* are multifactorial, unpredictable, and can occur at any stage of the manuscript lifecycle. *JMLA* has not been immune from the structural challenges in identifying peer reviewers that has been discussed at length by others within scholarly publishing [2–4]. Timing can be capricious too—a manuscript that is deemed ready for publication may be accepted right before or right after a full issue is sent to production, which results in a manuscript heading to production within a couple of months of acceptance or being delayed for another three months. Nor are we, the editorial team, blameless. As an MLA membership-supported, diamond open access journal, *JMLA*'s editorial team is entirely volunteer. Many times, other professional or personal demands on the editors' time pull our attention elsewhere, delaying manuscripts at different stages throughout the process that otherwise might move forward more quickly.

However, in our experience, one of the most influential variables in time-to-publish can be the authors. *JMLA* has operated under a “revise-at-will” workflow, where authors are given as much time as they would like to submit a revised manuscript at each stage of publication. How authors respond to this autonomy differs considerably, with some authors revising manuscripts within a couple of business days, while others never submit a revised manuscript at all.

The editorial team at *JMLA* has made the decision to change our “revise-at-will” policy in favor of firmer deadlines. Beginning with this issue, manuscripts returned to authors with a “revise and resubmit” decision must submit their revised submission within two months of the editorial decision. Likewise, manuscripts returned to authors with a “revisions required” decision must be resubmitted within one month of the editorial decision. Mutually agreeable accommodations to reasonably extend these deadlines on a case-by-case basis can be done if the author(s) and editor(s) are engaged in communication throughout the process.

We did not institute this policy change lightly. We recognize that the majority of *JMLA*'s core audience of authors and readers are information science practitioners, for whom publishing is not a primary responsibility of their position [5]. We also understand the longstanding, structural barriers that librarians encounter when trying to engage in publishing and scholarship [6,7]. Given our awareness of those barriers, it might seem frustrating to institute a deadline policy that appears to rachet up the pressure to publish even more acutely. However, we feel confident this decision will improve the publishing experience for everyone in *JMLA*'s community of editors, reviewers, and authors and give better predictability to the time-to-publication question that is often asked.

These new deadlines will prevent the significant disruptions the “revise-at-will” policy creates in *JMLA*'s production process. A more predictable timeline will allow our entirely volunteer editorial team to better schedule time to handle the manuscripts. Currently, we struggle to balance our work at *JMLA* against our other responsibilities because we cannot know with any confidence when (if ever) manuscripts will be returned and need our attention. With clearer deadlines, the editorial team will also be able to create *JMLA* issues that are better balanced in terms of extent and topics covered. Our colleagues within the MLA staff who serve as our copy and production editors will also be able to plan their workflows, avoiding the intensive crunch periods they currently experience at publication time.

Removing these lengthy delays will also create more reasonable expectations of our peer reviewers, whose uncompensated contributions to the journal are essential to its continued success [8]. Under our current “revise-at-will” model, we often must ask reviewers to take a second look at a “revise and resubmit” manuscript they last read more than six months ago. When dealing with such significant lag times between original submission and resubmission, it is unreasonable to expect reviewers to recall even the broad points of the manuscript, much less the specific and enumerated comments they shared with the authors originally. A shorter two-month timeline will enable our reviewers to provide more thoughtful commentary to authors who decide to undertake a full revision of their original submission.

Finally, while the latitude afforded by the “revise-at-will” policy appeared more accommodating for our authors, we suspect this policy was not in our authors' best interests. Research on academic writing suggests that creating structures and accountability (e.g., writing schedules, writing accountability groups, and externally set deadlines) can help writers overcome procrastination and enhance their productivity [9–11].

We anticipate this additional structure and guidance will help more prospective authors achieve their goal of seeing their work published in our journal while improving our ability to estimate timelines and keep production on schedule. As with our recently introduced policy on the use of generative AI [12], this policy will evolve according to the needs of our community.
